# Size Enlargement Enabled Functional Profiling of Extracellular Vesicle at Single-Particle Level

**DOI:** 10.3390/bios16040230

**Published:** 2026-04-21

**Authors:** Jia Yao, Xianyue Ji, Xingyu Tao, Ziyan Li, Shao Su, Xianguang Ding

**Affiliations:** State Key Laboratory of Flexible Electronics (LoFE), Institute of Advanced Materials (IAM), Nanjing University of Posts and Telecommunications, Nanjing 210023, China

**Keywords:** macrophages, extracellular vesicles, heterogeneity

## Abstract

Extracellular vesicles (EVs) are promising biomarkers for liquid biopsy, but their clinical application is limited by intrinsic heterogeneity and the lack of methods capable of resolving functionally distinct EV subpopulations at the single-vesicle level. Conventional bulk analyses obscure rare but clinically relevant EV subsets, while most single-EV approaches focus on physical properties or surface markers, with limited access to intravesicular functional information. Here, we report a fusion-enabled EV detection strategy at the single-particle level for functional profiling of macrophage-derived EVs. Liposomal probes encapsulating L-arginine, NADPH, and a nitric oxide (NO)-responsive fluorescent dye are engineered to fuse with EV membranes, delivering substrates into the vesicle lumen. In macrophage-derived EVs, inducible nitric oxide synthase (iNOS) catalyzes NO production, activating the fluorescent probe and generating a localized signal within individual vesicles. Signal generation is confined to vesicle-restricted reactions, ensuring specificity and minimizing background. The formation of hybrid vesicles further facilitates optical detection using conventional fluorescence microscopy.

## 1. Introduction

Liquid biopsy encompasses a range of circulating biomarkers, including circulating tumor DNA (ctDNA), circulating tumor cells (CTCs), cell-free RNA (cfRNA), and extracellular vesicles (EVs), each providing complementary information about disease status. ctDNA enables sensitive detection of genetic alterations but is often fragmented and present at low abundance, particularly in early-stage disease. CTCs provide intact cellular material and phenotypic information but are rare and technically challenging to isolate. Cell-free RNA offers insights into transcriptional dynamics but is susceptible to rapid degradation. In comparison, EVs are membrane-enclosed vesicles that protect their molecular cargo and are actively secreted by cells, allowing them to reflect dynamic cellular states and intercellular communication. These features make EVs an attractive target for liquid biopsy. Despite this potential, the clinical application of EVs is hampered by their inherent heterogeneity [1,2,3]. A primary challenge is distinguishing specific EV subpopulations, such as those derived from tumors, from the vast background of non-target EVs released by normal cells. This task is complicated by several factors: the low abundance of EVs derived from small primary tumors, the fact that not all tumor-derived EVs carry specific tumor biomarkers, and the overlapping physical characteristics (e.g., size, morphology) among different EV subtypes [4,5]. Conventional bulk analysis methods, such as Western blotting, enzyme-linked immunosorbent assay (ELISA), and reverse transcription-quantitative polymerase chain reaction (RT-qPCR), while standard for measuring EV cargo, require large sample volumes (typically >500 µL) to isolate sufficient EVs [6]. Moreover, these methods only provide an averaged signal from the entire EV population, masking underlying heterogeneity and making the detection of rare but clinically significant EV subpopulations impractical [7].

Single-EV analysis has emerged as a powerful strategy to resolve EV heterogeneity beyond bulk measurements. A variety of approaches have been developed, including fluorescence labeling, immunoaffinity-based detection of surface proteins, and nucleic acid profiling using amplification strategies [8,9,10]. These methods have enabled sensitive and multiplexed analysis of EV-associated biomolecules at the single-particle level. However, most existing techniques primarily focus on physical characteristics or molecular composition, such as surface markers or nucleic acid cargo, while providing limited access to intravesicular functional activity [11,12]. In particular, direct readout of enzymatic processes occurring within EVs remains challenging, as it typically requires access to the vesicle lumen without disrupting membrane integrity. Therefore, developing strategies that enable functional interrogation of EV-associated activity at the single-particle level represents an important complement to current molecular profiling approaches [13]. A key requirement for robust single-EV analysis is a stable and amplifiable signal output, often contingent on the efficiency of immunolabeling and the localization of target molecules. Among various signal amplification strategies, such as branched DNA probes or enzymatic reactions, plasmon-enhanced fluorescence has demonstrated powerful multiplexed signal amplification. While imaging techniques like high-resolution CT and MRI are valuable, their sensitivity for detecting minimal residual disease can be limited [14,15,16,17].

**Figure 1:**
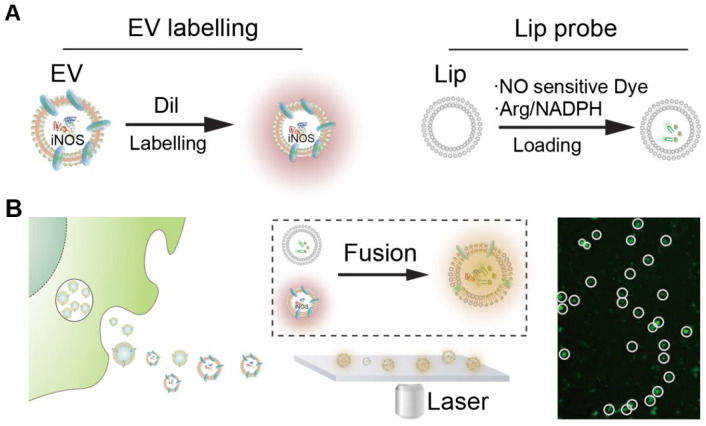
Schematic illustration of the single extracellular vesicle (EV) detection strategy. (**A**) Macrophage-derived EVs are labeled with the lipophilic dye Dil, producing a red–orange membrane fluorescence signal. In parallel, liposomal probes are prepared by encapsulating L-arginine, NADPH, and a nitric oxide (NO)-sensitive fluorescent dye; (**B**) Following membrane fusion between macrophage-derived EVs and liposomal probes, intravesicular iNOS catalyzes NO production, activating the NO-sensitive dye. Co-localized detection of Dil fluorescence (EV membrane) and NO-induced green fluorescence enables functional identification of single EVs based on catalytic activity.

In this study, we introduce a single-EV detection strategy that exploits the intrinsic activity of inducible nitric oxide synthase (iNOS) in macrophage-derived EVs. The method employs engineered liposomal probes encapsulating L-arginine, NADPH, and a nitric oxide (NO)-responsive fluorescent dye. Through membrane fusion, these substrates are delivered into EVs, enabling intravesicular iNOS to catalyze NO production and activate fluorescence. This fusion-enabled enzymatic reaction generates a localized and amplified signal at the single-vesicle level (Figure 1). In addition, the formation of larger hybrid vesicles facilitates optical detection. We demonstrate that this platform enables functional profiling of EV subpopulations and supports applications in disease-related EV analysis. In addition, the use of conventional fluorescence microscopy for signal readout improves accessibility compared with specialized instrumentation required for some advanced single-EV platforms.

## 2. Materials and Methods

### 2.1. Materials and Reagents

The nitric oxide (NO) fluorescent probe kit, cell membrane extraction reagent, bicinchoninic acid (BCA) protein assay kit, and lipopolysaccharide (LPS) were purchased from Beyotime (Shanghai, China). Antibodies against CD63, CD80, and CD206, were purchased from Biolegend (San Diego, CA, USA). The small molecule inhibitor BLZ945 was sourced from Aladdin (Shanghai, China). Fluorescence measurements were performed using a fluorescence spectrophotometer (Horiba, Kyoto, Japan). Absorbance readings for assays were taken with a microplate reader (BioTek, Winooski, VT, USA). Cell and vesicle imaging were conducted using an inverted fluorescence microscope (Olympus, Tokyo, Japan). Statistical analysis was performed using the software GraphPad 8.0. All data were shown as mean values ± s.d.

### 2.2. Cell Culture

The mouse macrophage cell line RAW 264.7 was used in this study (RAW264.7 cells were commercially obtained from the American Type Culture Collection (ATCC), Manassas, VA, USA. Catalog Number: TIB-71). Cells were cultured in RPMI 1640 medium containing 10% (*v*/*v*) heat-inactivated fetal bovine serum (FBS) and 1% (*v*/*v*) penicillin–streptomycin antibiotic solution. All cells were grown in a humidified atmosphere of 5% CO_2_ at 37 °C.

### 2.3. Preparation of Liposomal Probes

Liposomal probes were prepared using a thin-film hydration and extrusion method. Briefly, 1 mg of phosphatidylcholine was dissolved in 9 mL of a chloroform: methanol (6:3, *v*/*v*) mixture. The organic solvent was evaporated using a rotary evaporator to form a thin lipid film. The film was hydrated with 1 mL of phosphate-buffered saline (PBS) containing the nitric oxide (NO)-sensitive fluorescent dye (5 mM, Beyotime Biotechnology), 40 μL of L-arginine solution, and 30 μL of NADPH (0.1 mM). The mixture was sonicated to disperse the lipids and then extruded through a polycarbonate membrane with a pore size of 800 nm to obtain unilamellar vesicles. The resulting liposomal suspension was stirred for 1 h and subsequently centrifuged at 18,047× *g* for 30 min at 4 °C. The supernatant was discarded, and the pellet containing the purified liposomal probes was resuspended in PBS, aliquoted, and stored at 4 °C for future use.

### 2.4. Isolation and Labeling of EVs

EVs were isolated from cell culture supernatants by differential centrifugation. When macrophages reached approximately 80% confluency, the culture medium was replaced with serum-free medium for 24 h. The conditioned medium was collected and sequentially centrifuged at 500× *g* to remove cells and large debris, followed by 10,000× *g* for 90 min to eliminate larger vesicles and organelles. The resulting supernatant was subjected to ultracentrifugation at 100,000× *g* for 2 h at 4 °C to pellet the EVs. The supernatant was discarded, and the EV pellet was washed and labeled with the lipophilic dye DiI (10 μM) for 20 min at 37 °C. Unincorporated dye was removed by a final ultracentrifugation step. The purified, labeled EV pellet was resuspended in phosphate-buffered saline (PBS), aliquoted, and stored at −80 °C for subsequent experiments.

### 2.5. Membrane Fusion Assay

The membrane fusion reaction was initiated by mixing EVs with liposomal probes at an input ratio of 1:2 (EVs:liposomal probes). The mixture was acidified to pH 5.0 and incubated at 37 °C for 2 h. The samples were sufficiently diluted to allow particle fusion at the single-particle level. Following incubation, the fluorescence signal was measured using a fluorospectrophotometer with an excitation wavelength of 495 nm and an emission wavelength of 515 nm. A sample was considered positive if its fluorescence intensity exceeded the cutoff threshold, defined as the mean signal of the negative control plus three times its standard deviation.

## 3. Results

### 3.1. Assay Design and Detection Principle

To enable detection of extracellular vesicles (EVs) at the single-particle level without introducing marker bias, EV membranes were uniformly labeled with the lipophilic dye Dil. This signal serves as a reference for identifying the total EV population. The detection strategy is based on coupling membrane fusion with enzymatic signal generation. Liposomal probes were engineered to encapsulate L-arginine, NADPH, and a nitric oxide (NO)-responsive fluorescent dye. Upon fusion with EVs, these substrates are delivered into the vesicle lumen, where inducible nitric oxide synthase (iNOS), if present, catalyzes the production of NO. The generated NO subsequently activates the fluorescent probe, producing a localized signal. A valid detection event is defined by the co-localization of DiI fluorescence and NO-dependent fluorescence, indicating both successful fusion and the presence of functional iNOS within the same vesicle. This dual-signal design reduces ambiguity associated with nonspecific probe activation and enables identification of functionally active EV subpopulations. In addition, fusion with relatively large liposomal probes (~800 nm) increases the effective size of the vesicles, facilitating direct visualization of individual EVs using conventional fluorescence microscopy.

**Figure 2:**
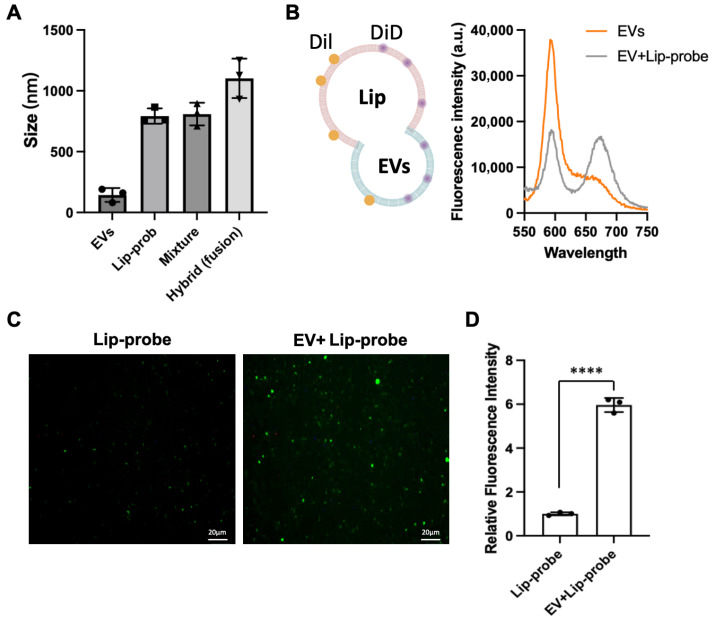
Validation of membrane fusion and enzymatic signal generation. (**A**) Dynamic light scattering (DLS) analysis of EVs, liposomal probes (Lip-prob), their mixture before fusion, and the resulting hybrid particles after fusion. The increase in particle size after fusion is consistent with the formation of enlarged EV–liposome hybrid vesicles. (**B**) Schematic illustration of the FRET-based membrane fusion assay and corresponding fluorescence spectra. EVs and liposomal probes were labeled with DiI and DiD, respectively. Following fusion, excitation of DiI resulted in decreased donor emission and increased DiD acceptor emission, consistent with membrane fusion–induced fluorophore proximity. (**C**) Representative fluorescence images of liposomal probes (Lip-probe) and EV–liposome mixtures (EV + Lip-probe). A marked increase in green fluorescence is observed only in the presence of EVs, indicating fusion-dependent activation of the NO-responsive probe. Scale bar: 20 μm; (**D**) Quantitative analysis of fluorescence intensity corresponding to panel (**C**), demonstrating significantly enhanced signal following EV-liposome fusion. Data are means ± SD, n = 3 for each group, biologically independent samples. The significant differences were calculated based on one-way ANOVA. **** *p* < 0.0001.

We first sought to confirm that membrane fusion occurs efficiently and leads to functional signal generation. Dynamic light scattering (DLS) analysis revealed that the average particle size increased to approximately 1000 nm after incubation compared to free EVs, Lip-prob and mixture of EVs and Lip-prob before fusion (Figure 2A), consistent with the formation of hybrid vesicles from EVs (50–200 nm) and liposomal probes (~800 nm). To further substantiate membrane fusion, extracellular vesicles (EVs) and liposomal probes (Lip-prob) were labeled with DiI and DiD, respectively, to evaluate whether fluorescence resonance energy transfer (FRET) occurs upon merging of the two membrane structures. Following fusion, excitation at 549 nm (the excitation wavelength of DiI) resulted in a marked decrease in DiI fluorescence accompanied by a pronounced increase in DiD emission (Figure 2B). This spectral shift arises from the effective fusion of the two membranes, which brings DiI and DiD into close spatial proximity, thereby enabling FRET. These results collectively indicate the formation of stable hybrid structures rather than nonspecific aggregation.

Fluorescence microscopy provided direct evidence of enzymatic signal generation. Distinct green fluorescent puncta were observed in the EV–liposome mixtures (Figure 2C), each corresponding to an individual vesicle exhibiting NO-dependent fluorescence. Since probe activation requires NO production, these signals indicate that substrates were successfully delivered into the EV lumen and utilized by intravesicular iNOS. Quantitative analysis further demonstrated that the fluorescence intensity of the fusion system was significantly higher than that of liposomal probes alone (Figure 2D), confirming that background activation of the probe is minimal. The discrete nature of the fluorescence signals also suggests that the reaction occurs within confined vesicular compartments rather than in bulk solution. A small number of non-specific fluorescent events lacking co-localization with DiI were observed during initial experiments. These signals were minimized by optimizing fusion conditions, particularly reaction time and pH, guided by the emergence of the ~1000 nm particle population.

Together, these results confirm that liposomal probes can effectively fuse with EV membranes, deliver functional substrates, and generate a localized fluorescence signal dependent on intravesicular enzymatic activity.

### 3.2. Discrimination of EV Subtypes

**Figure 3:**
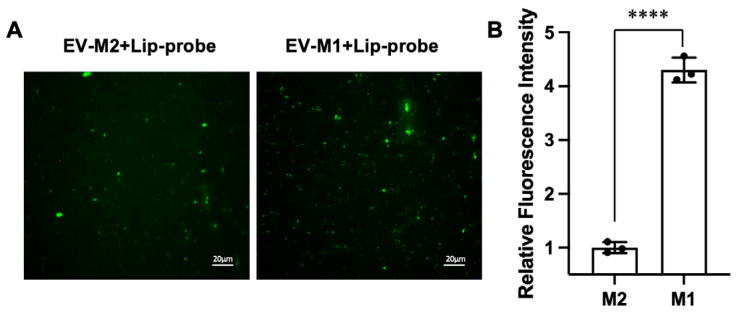
Discrimination of EV subpopulations based on iNOS activity. (**A**) Representative fluorescence images of EVs derived from M2- and M1-polarized macrophages after fusion with liposomal probes. Enhanced green fluorescence is observed in M1-derived EVs, indicating higher iNOS activity compared to M2-derived EVs. Scale bar: 20 μm; (**B**) Quantitative analysis of fluorescence intensity corresponding to panel (**A**), showing significantly increased signal in M1-EVs relative to M2-EVs. Data are means ± SD, n = 3 for each group, biologically independent samples. The significant differences were calculated based on one-way ANOVA. **** *p* < 0.0001.

To evaluate whether the platform can resolve biologically distinct EV populations, EVs derived from polarized macrophages were analyzed. In this assay, EV-associated particles were identified based on the DiI membrane label, and functional readout was assigned only to puncta showing co-localized DiI and NO-dependent fluorescence after fusion. Using this criterion, EVs from M1 macrophages exhibited significantly higher fluorescence intensity than those from M2 macrophages (Figure 3A,B), consistent with the known upregulation of iNOS in the M1 phenotype. The mean fluorescence intensity of M1-derived EV-associated particles was approximately 1.8-fold higher than that of the M2 group. In addition to differences in average intensity, fluorescence imaging revealed a higher number of dual-positive particles in the M1 group, indicating an increased proportion of EVs carrying functional iNOS activity. This observation suggests that the method captures not only changes in enzyme activity but also shifts in the distribution of functionally active EV subpopulations. Together, these results support that the assay can distinguish biologically distinct EV subtypes based on EV-associated enzymatic activity at the single-particle level.

**Figure 4:**
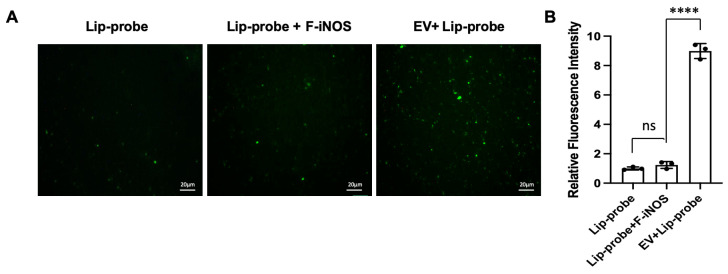
Specificity of the membrane fusion assay. (**A**) Representative fluorescence images of liposomal probes alone (Lip-probe), liposomal probes incubated with free iNOS (Lip-probe + F-iNOS), and EV–liposome mixtures (EV + Lip-probe). Strong fluorescence is observed only in the EV + Lip-probe group, while minimal signal is detected in the presence of free iNOS, indicating that probe activation requires membrane fusion. Scale bar: 20 μm; (**B**) Quantitative analysis of fluorescence intensity corresponding to panel (**A**), showing significantly enhanced signal exclusively in EV-containing samples. Data are means ± SD, n = 3 for each group, biologically independent samples. The significant differences were calculated based on one-way ANOVA. ns, no siginificant difference. **** *p* < 0.0001.

To determine whether fluorescence generation is specific to EV-associated iNOS, control experiments were performed using free iNOS under identical conditions. The mixture of liposomal probes with free iNOS produced only weak fluorescence, comparable to background levels (Figure 4A,B). In contrast, EV samples generated a substantially stronger signal, approximately an order of magnitude higher than that observed with free enzyme (Figure 4A,B). This difference indicates that signal generation requires confinement of the enzymatic reaction within the vesicular environment, which is achieved through membrane fusion. In the absence of fusion, encapsulated substrates remain inaccessible to enzymes in solution, preventing nonspecific signal generation.

**Figure 5:**
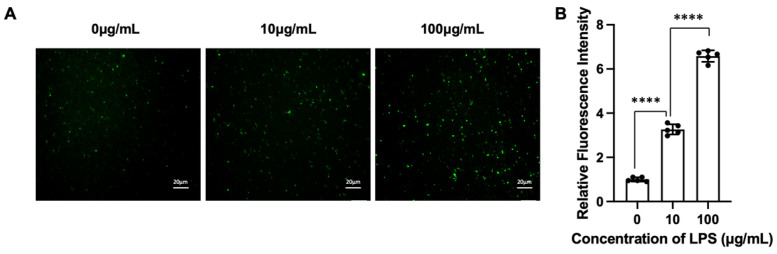
Dose-dependent response of EV-associated iNOS activity to LPS stimulation. (**A**) Representative fluorescence images of EVs isolated from macrophages treated with increasing concentrations of lipopolysaccharide (LPS; 0, 10, and 100 μg/mL) after fusion with liposomal probes. Increased fluorescence intensity is observed with higher LPS concentrations, indicating elevated iNOS activity in EVs. Scale bar: 20 μm; (**B**) Quantitative analysis of fluorescence intensity corresponding to panel (**A**), showing a concentration-dependent increase in signal. Data are means ± SD, n = 3 for each group, biologically independent samples. The significant differences were calculated based on one-way ANOVA. **** *p* < 0.0001.

We next evaluated the sensitivity of the assay using EVs derived from macrophages stimulated with increasing concentrations of lipopolysaccharide (LPS). A clear dose-dependent increase in fluorescence intensity was observed (Figure 5), accompanied by an increase in the number of fluorescent vesicles. This trend reflects an elevated proportion of iNOS-positive EVs under stronger inflammatory stimulation. Together, these results demonstrate that the assay achieves both high specificity, by restricting signal generation to fusion-mediated events, and sufficient sensitivity to detect biologically relevant changes in EV populations.

### 3.3. Single-EV Analysis

To visually confirm the detection capability of our system at the single-vesicle level, the post-fusion solution was applied to a glass slide and examined under an inverted fluorescence microscope. Individual EVs appeared as distinct fluorescent puncta distributed across the image. The analysis of co-localization was performed on EVs dually labeled with Dil (red-orange, membrane marker) and the NO-sensitive fluorescent probe (green, indicative of iNOS activity) following the fusion assay.

**Figure 6:**
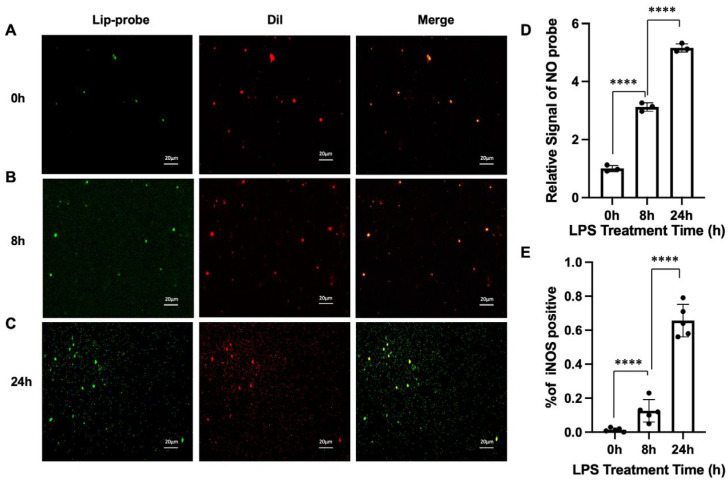
Dynamic monitoring of macrophage repolarization via EV-associated iNOS at the single-vesicle level. (**A**–**C**) Representative fluorescence images of EVs derived from macrophages after 0 h (**A**), 8 h (**B**), and 24 h (**C**) of LPS stimulation following fusion with liposomal probes. EV membranes are labeled with Dil (red), and iNOS activity is indicated by NO-responsive fluorescence (green). Co-localization appears as yellow puncta in merged images, representing iNOS-positive EVs. Scale bar: 20 μm; (**D**) Quantitative analysis of NO-dependent fluorescence intensity at different time points, showing a time-dependent increase in signal; (**E**) Quantification of the proportion of iNOS-positive EVs, defined as the fraction of co-localized puncta relative to total Dil-labeled EVs, demonstrating an increasing trend with prolonged LPS stimulation. Data are means ± SD, n = 3 for each group, biologically independent samples. The significant differences were calculated based on one-way ANOVA (**D**,**E**). **** *p* < 0.0001.

We observed a subset of EVs within the population that exhibited both red and green fluorescence. The co-localization of these signals within the same EV particle, visible as yellow puncta in merged images (Figure 6A–C), confirmed the presence of functional iNOS. To quantify this, the fluorescence images from both channels were converted to grayscale. After identifying EV regions based on the Dil signal, the fluorescence intensity inside and outside these regions was calculated. The overlapping area between the two images was defined as an iNOS-positive EV. The merged images revealed that the intensity of the green fluorescence (iNOS signal) increased with the duration of LPS stimulation (Figure 6D), indicating a time-dependent upregulation of iNOS in EVs during macrophage repolarization. This signal was detectable even under varying conditions, demonstrating the high sensitivity of our system. We further quantified the proportion of iNOS-positive EVs by calculating the ratio of dually labeled EVs (co-localized puncta) to the total Dil-labeled EV population within a field of view using ImageJ 1.54q software. This proportion, representing the fraction of EVs containing functional iNOS, increased significantly with longer LPS stimulation times (Figure 6E).

**Figure 7:**
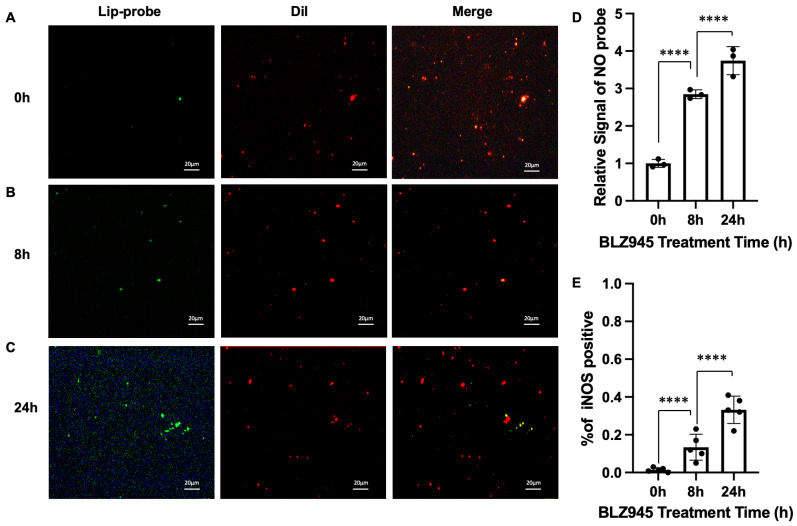
Monitoring macrophage polarization dynamics induced by BLZ945 using the single-EV fusion assay. (**A**) Fluorescence image of the fusion assay with EVs derived from macrophages without BLZ945 treatment (control group). EVs are labeled with Dil (red). Scale bar, 20 μm; (**B**) Assay image with EVs obtained after 8 h of BLZ945 stimulation; (**C**) Assay image with EVs obtained after 24 h of BLZ945 stimulation. The merged image shows yellow puncta, indicating the co-localization of Dil (EV membrane) and the NO probe (iNOS activity), which signifies successful fusion and detection of iNOS-positive EVs; (**D**) Quantitative analysis of the relative signal intensity of NO at different treatment time points (0 h, 8 h, 24 h); (**E**) Quantitative analysis of the signal intensity or the proportion of iNOS-positive EVs across the different treatment time points (0 h, 8 h, 24 h). Data are means ± SD, n = 3 for each group, biologically independent samples. The significant differences were calculated based on one-way ANOVA (**D**,**E**). **** *p* < 0.0001.

To validate the general applicability of this detection method, we repeated the experiment using BLZ945 as an alternative polarizing agent. A similar dose- and time-dependent increase in iNOS-positive EVs was observed (Figure 7D,E), confirming the robustness of our assay. However, the fluorescence intensity and the proportion of iNOS-positive EVs were higher in LPS-treated samples compared to BLZ945-treated ones at equivalent time points, suggesting that LPS induces a stronger inflammatory polarization response. In conclusion, our method provides a versatile and highly effective platform for the dynamic monitoring of functional cargo within individual EVs, enabling precise analysis of heterogeneous EV populations.

## 4. Discussion

While the present study demonstrates the feasibility of fusion-enabled functional analysis of EV-associated enzymatic activity, several limitations and sources of uncertainty should be considered. First, membrane fusion is a central step in the assay, yet fusion efficiency and its variability were not quantitatively determined in this study. The current system was established under empirically optimized conditions, and the extent to which individual EVs undergo fusion or receive sufficient substrate for signal generation may vary, potentially contributing to signal heterogeneity. Second, the assay relies on enzymatic activation of a fluorescent probe, which introduces potential sources of false-positive and false-negative signals. For example, incomplete fusion or insufficient substrate delivery may lead to underestimation of active EV populations, while nonspecific probe activation or background fluorescence could contribute to overestimation if not properly controlled. In this work, EV-associated signals were defined based on co-localization with a membrane label, which provides an operational criterion but does not fully exclude all non-specific contributions.

The current implementation focuses on iNOS activity in macrophage-derived EVs as a model system, and the broader applicability of this platform to other types of EV-associated cargo or disease contexts remains to be established. EV populations exhibit substantial biological variability in cargo composition and functional activity, and not all EVs may contain enzymatically active components that can be readily interrogated using this strategy. Extending the approach to other enzymes or functional biomarkers will require careful selection of probe chemistry and validation of signal specificity. Therefore, while the present results demonstrate a functional readout capability at the single-particle level, further work will be needed to assess the generalizability and robustness of the method across diverse biological systems. Taken together, the present study provides a proof-of-concept demonstration of a fusion-enabled strategy for probing EV-associated functional activity at the single-particle level. The method complements existing composition-based EV analysis approaches by introducing an activity-based dimension, but it should be interpreted within the limitations of the current experimental framework. Future efforts will be required to improve quantitative control of fusion, reduce background signals, and evaluate performance in more complex biological samples before broader analytical or clinical applications can be realized [18,19,20,21].

## 5. Conclusions

Liquid biopsy enables non-invasive monitoring of tumor evolution, offering significant advantages over traditional tissue biopsy, which requires invasive procedures such as surgery or bronchoscopy to obtain diagnostic material. Extracellular vesicles (EVs) carry a rich cargo of biomolecules, including nucleic acids (e.g., mRNAs and miRNAs) and proteins, that are derived from their parent cells, which makes them excellent sources of disease biomarkers. Numerous studies have demonstrated that EVs can mirror tumorigenesis and disease progression, thereby solidifying their status as one of the most promising biomarkers for liquid biopsy. However, the small size and heterogeneous nature of EVs pose significant challenges for extracting sufficient clinical information using conventional methods. Conventional methods for analyzing proteins and nucleic acids, such as Western blotting and RT-qPCR, typically require substantial amounts of sample. Consequently, reliable techniques for measuring the expression of specific proteins and nucleic acids at the single-EV level are still lacking.

EVs are intrinsically heterogeneous in size, cargo, and function owing to their diverse cellular origins. Current EV-based liquid biopsy approaches typically involve bulk analysis of heterogeneous EV populations, which yields an averaged signal that masks the characteristics of individual EV subtypes. Therefore, analysis at the single-EV level can provide more detailed information, such as revealing heterogeneity in biomarker expression and enabling the identification of distinct EV subpopulations. This approach is crucial for identifying disease-specific EVs, as the biomarkers they carry are upregulated or downregulated in response to pathological changes, highlighting their significant value for early disease detection.

For single-EV analysis, while surface proteins can be directly detected using methods such as immunoassays, analyzing intravesicular components (e.g., RNA) requires accessing the EV lumen, often while preserving the vesicle’s structural integrity. Previous strategies often involved lysing EVs to release their contents for subsequent analysis. However, this lysis-based approach is problematic because the detergents used to permeabilize or dissolve the EV membrane can cause the loss of target biomarkers. Moreover, lysis efficiency is highly dependent on factors such as EV subtype, detergent type and concentration, and buffer conditions (e.g., incubation time and temperature), inevitably introducing variability and potential inaccuracies. In contrast, our membrane fusion-based detection strategy effectively circumvents these limitations.

## Data Availability

The raw and processed data required to reproduce these findings are available from the corresponding author upon reasonable request.
